# Effects of Acupuncture on Th1, Th2 Cytokines in Rats of Implantation Failure

**DOI:** 10.1155/2012/893023

**Published:** 2012-01-23

**Authors:** Juan Gui, Fan Xiong, Jing Li, Guangying Huang

**Affiliations:** Institute of Integrated Traditional Chinese and Western Medicine, Tongji Hospital, Tongji Medical College, Huazhong University of Science and Technology, Wuhan, Hubei 430030, China

## Abstract

The aim is to explore the effect of acupuncture on Th1, Th2 cytokines in rats of implantation failure. Early pregnant rats were randomized into normal group (N), implantation failure group (M), acupuncture group (A), progestin group (H). The model was established with mifepristone. Samples of serum, endometrium were collected on Day 5, 6 and 8 of pregnancy. Compared with group M, the number of embryos was significantly higher in groups N, A and H; IL-1**β**, IL-2 protein in serum and endometrium were significantly lower in groups N, A and H, while IL-4, IL-10 were significantly higher in groups N, A and H; the endometrial IL-2, IL-4 mRNA were significantly lower in groups N, A and H, while IL-1**β**, IL-10 mRNA were significantly higher in groups N, A and H. Acupuncture could improve the poor receptive state of endometrium due to mifepristone by promoting Th2 cytokines secretion and inhibiting Th1 cytokines to improve blastocyst implantation.

## 1. Introduction

In recent years, the assisted reproductive technology has been greatly improved, but the success rate is still not high. The primary reason is blastocyst implantation failure. Implantation is one of the most important procedures in reproduction and the key for the success of pregnancy. In the process of blastocyst implantation, in order to adapt to the blastocyst adhesion, intrusive, growth, and development, the endometrium suffers complex changes including cell proliferation, differentiation, migration, and apoptosis. Various molecules are participating in this process. Thus, the crosstalk between the active blastocyst and receptive uterus is essential to implantation [1]. It is crucial to take timely modifications in the endometrium to become receptive to the developing embryo for successful implantation. [2]. The human endometrium becomes receptive to the embryo only for a limited period during the luteal phase of the menstrual cycle, under the influence of steroid hormones and paracrine factors originating from endometrial cells and the embryo [3]. Impaired endometrial receptivity is considered to be a major limiting factor for the establishment of pregnancy [4]. The regulation of endometrial changes is not exclusively by ovarian hormones, the immune system has been implicated in normal endometrial function, similar to processes taking place during inflammatory and reparative path. Thus, cytokines and immune cells play a major role in endometrial tissue regeneration, growth, differentiation, and shedding throughout the normal menstrual cycle and in remodelling during embryonic implantation and growth [5–7]. 

Cytokines play a critical role in pregnancy, particularly in the early stages during blastocyst implantation and placental development [8–13]. For some years, a prevalent theory has been that predominant production of so-called T helper type 2 (Th2) cytokines such as interleukin (IL)-4 and IL-10 was characteristic of normal implantation and pregnancy, whereas in miscarriage, and recurrent miscarriage there was a predominant production of Thl cytokines such as IL-1 and IL-2 [14]. Therefore in the process of location, adhesion, and penetration, the endometrium not only has the morphologic changes but also has alteration in the expression of cytokines.

Repeated failures of in vitro fertilization and embryo transfer (IVF-ET) bring huge economic and psychological burden to infertility patients, therefore, we are expecting an economic and effective method to ameliorate implantation. A research has shown that acupuncture on the day of ET significantly improves the reproductive outcome of IVF, compared with no acupuncture [15]. Acupuncture seems to be a useful tool for improving pregnancy rate after assisted reproduction therapy [16]. Previous researches have proven that acupuncture treatment is effective for blastocyst implantation obstacle, but the mechanism is still not clear, so the aim of the experiment is to explore the possible way that acupuncture improves implantation and to provide more supports for rationale.

## 2. Materials and Methods

### 2.1. Animals and Grouping

Virgin, 10-week-old, weight 210 ~ 230 g female Wistar rats (*n* = 90) and reproductive adult male Wistar rats (*n* = 40), weight 250 ~ 300 g, SPF grade were provided by the CDC of Hubei province (the animal certificate SCXK no. 2008-0005) and fed in the barrier system according to the institutional guidelines established by the Animal Care and Use Committee of Tongji Medical College, Huazhong University of Science and Technology. After adaptive feeding for 5 days, female rats were mated with male rats at 6 PM with the scale of 2 : 1 and checked the vaginal smear at 8 AM the next day (D1 = sperm on the vaginal smear detection). The pregnant rats were randomized into normal group (N), implantation failure group (M), acupuncture treatment group (A), and progestin treatment group (H), with 18 rats in each group. Then, the 18 rats were equally randomized into D5 group (*n* = 6), D6 group (*n* = 6), D8 group (*n* = 6) according to the time of sampling.

### 2.2. Reagent and Main Devices

The mifepristone tablets (Beijing Zizhu Pharmaceutical Co., Ltd. China) and progestin (Zhejiang Xianju Pharmaceutical Co., Ltd. China) were provided by Tongji Hospital. DAB coloring reagent kit used for immunohistochemistry was product of Beijing Zhongshan Biotech Co., Ltd. China. Tissue protein extraction (AR0101-100) and BCA protein assay kits (AR0146) were purchased from Wuhan BOSTER Company, China. Cocktail protease inhibitor was purchased from Wuhan Gugeshengwu Technology Co., Ltd. China. Rat IL-1*β*, IL-2, IL-4, and IL-10 enzyme-linked immunosorbent assay (ELISA) kits were purchased from Beijing NeoBioscience Technology Co., Ltd. China. The total RNA extract reagent (Trizol), PrimeScript RT reagent Kit Perfect Real Time (TaKaRa Code: DRR037A), and SYBR Premix Ex Taq (TaKaRa Code: DRR041) were purchased from TaKaRa Biotechnology (Dalian) Co., Ltd. Dalian, China. Rabbit polyclonal to IL-1 beta (ab9787), IL-2 (ab25104), IL-4 (ab9811), and IL-10 (ab9969) was purchased from Abcam Company, USA. Goat or rabbit anti-rat Actin polyclonal antibody (sc-1616R) was purchased from Santa Cruz Biotechnology, Inc. California, USA. DyLight 800-Labeled Antibody To Rabbit IgG (H + L) (Cat. No 072-07-15-06) was purchased from KPL, Inc. Maryland, USA. HRP-Goat anti-Rabbit IgG (H + L) Conjugate (Cat. No. ANT011) was purchased from AntGene Biotech Co., Ltd. China. Nucleic Acid/Protein Analyzer (DU730, BECKMAN COULTER, Inc., Fullerton, California, USA); Mastercycler gradient PCR apparatus (Eppendorf Company, Germany); Canon Micro-imaging System (Canon 350 D, Japan); Applied Biosystems StepOne Real-Time PCR System (Applied Biosystems, California, USA); Microplate reader (BioTek Synergy2, Vermote, USA); Near infrared double-color laser imaging system (Odyssey LI-COR Inc, USA).

### 2.3. Modeling and Treatment

The mifepristone tablet was dissolved in an appropriate amount of edible sesame oil after tripsis to produce 2 mg/mL mifepristone solution. The rats in groups M, A, and H were given mifepristone solution at 5.5 mg/kg by neck subcutaneous injection on D1 at 9 AM, while group N were injected with corresponding sesame oil. Group A started acupuncture treatment which selected bilateral “Housanli” (ST 36) and “Sanyinjiao” (SP 6) as the acupoints [17] from D1 at 3 PM and the rats were fixed by self-made cloth bags. Acupuncture methods: continuous 25 min per day, rolling the needle every 5 min, and treatment for 5–8 days by the same investigator for all the rats in group A. Group N and M were fixed for 25 min at daily 3 PM for 5–8 days. Group H began progestin treatment (40 mg/kg/day, *im*) from D1 for 5–8 days.

### 2.4. Sampling

The rats were narcotized with 1% pentabarbital sodium by intraperitoneal injection at 4 PM on D5, D6, and D8 for each group. Uterus was taken out by laparotomy and flushed with normal saline. Part of the uterus was fixed in 4% paraformaldehyde solution for paraffin embedding, while other part was preserved in −80°C refrigerator for future use. Blood was drawn from aorta abdominalis. After centrifuging at 3000 r/min for 20 min, the serum was collected and stored in a −80°C refrigerator.

### 2.5. ELISA for IL-1*β*, IL-2, IL-4, and IL-10 Protein Expression in Serum and Endometrium

A quantitative sandwich enzyme immunoassay technique was used in accordance with the manufacturer's protocol. The sensitivity of the ELISA kits was 15 pg/mL. None of the samples examined had a cytokine level >2000 pg/ml. The inter-assay and intra-assay coefficients of variation of the ELISA kits were less than 9%.

### 2.6. Immunohistochemistry for Endometrial IL-1*β*, IL-2, and IL-4 Protein Detection

The paraffin slides were kept in oven at 60°C for 1 hour. Then, the sections were deparaffinized in xylene and rehydrated through grades of ethanol in distilled water, rinsed with PBS three times (5 min each). After that, antigens were retrieved by microwave processing with 10 mM Na-citrate (pH 6.0) at 92–98°C for 20 min. After natural cooling to room temperature, rinsed in PBS, endogenous peroxidase activity was quenched with 3% H_2_O_2_ in PBS for 10 min, followed by washing in PBS, three times, 5 min each. Sections were blocked with 1% Bovine Serum Albumin (BSA) for 30 min. Excess BSA was drained. These sections were then incubated in respective primary antibody overnight at 4°C in a humidified chamber. For the negative control, slide was incubated in PBS. Primary antibodies against IL-1*β*, IL-2, and IL-4 raised in rabbit were used at 1 : 50 dilution. After being rinsed in PBST (0.1% Tween-20 in PBS), sections were then incubated for 1 h at 37°C with HRP-goat anti-rabbit diluted 1 : 200 in PBS. After three washes for 5 min in PBST, the sections were incubated in substrate diaminobenzidine for 3–5 min until the color developed. Then, the sections were counterstaining with hematoxylin for 3 min. After brief washes in distilled water, the slides were dehydrated in grades of ethanol, cleared for 20 min in xylene. Pictures were taken by the Canon Micro-imaging System and analyzed with Image-Pro Plus 6.0 to measure average optical intensity (AOI).

### 2.7. Western Blot for Endometrial IL-1*β*, IL-4, and IL-10 Protein Detection

Total protein was extracted from the endometrial implantation site tissues which were homogenated and lyzed in Mammal tissue protein extraction reagent, supplemented with protease inhibitor cocktail and phenylmethylsulfonylfluoride (PMSF), then centrifuged at 13201g for 10 min at 4°C. The supernatants were collected to quantify the protein concentration with the BCA protein assay kit. Uterine extracts (100 *μ*g protein) were mixed with sample buffer, boiled for 10 min, and ran on a 12% SDS-PAGE gel (100 v, 2 h). Separated proteins on the gel were transferred to nitrocellulose membranes. The membranes were blocked with 5% non-fat-dry milk in PBST for 2 h at room temperature and incubated overnight at 4°C with antibodies to *β*-actin, IL-1*β*, IL-4, or IL-10 diluted 1 : 200, 1 : 50, 1 : 50, and 1 : 50, respectively. Following four washes with PBST (5 min each), the membranes were lucifugally incubated with the DyLight 800-Labeled Antibody To Rabbit IgG (H + L) diluted 1 : 10000 at room temperature for 1 h. After lucifugally washing with PBST, the membranes were detected by near infrared double-color laser imaging system. The pictures were analyzed with Image J to calculate the gray scale ratio of *β*-actin.

### 2.8. Real-Time PCR for Endometrial IL-1*β*, IL-2, IL-4, and IL-10 Expression

Total RNA was extracted from endometrial homogenized tissue with Trizol reagent according to the manufacturer's instructions. RNA purity and concentration were measured by Nucleic Acid/Protein Analyzer. 1 *μ*g of extracted total RNA was reverse transcribed with PrimeScript RT reagent Kit in accordance with the manufacturer's instructions. The cDNA was kept at −20°C prior to PCR amplification. Real-Time PCR reactions were performed in 48 well optical PCR plates using an Applied Biosystems StepOne Real-Time PCR System according to the manufacturer's instructions. 2^−ΔΔCT^ was used for analyzing the data. Primer sequence:

*β*-actin
  Forward primer: 5′-GGAGATTACTGCCCTGGCTCCTA-3′
  Reverse primer: 5′-GACTCATCGTACTCCTGCTTGCTG-3′
IL-1*β*
  Forward primer: 5′-GCTGTGGCAGCTACCTATGTCTTG-3′
  Reverse primer: 5′-AGGTCGTCATCATCCCACGAG-3′
IL-2
  Forward primer: 5′-GCAGCGTGTGTTGGATTTGAC-3′
  Reverse primer: 5′-GCTCATCATCGAATTGGCACTC-3′
IL-4 
  Forward primer: 5′-TGCACCGAGATGTTTGTACCAGA-3′
  Reverse primer: 5′-TTGCGAAGCACCCTGGAAG-3′
IL-10
  Forward primer: 5′-CAGACCCACATGCTCCGAGA-3′
  Reverse primer: 5′-CAAGGCTTGGCAACCCAAGTA-3′

### 2.9. Statistics

All data had a normal distribution presented as mean ± standard deviation (SD) and analysed by SPSS17.0 Statistical Software. Statistical significance was determined by one-way analysis of variance (ANOVA) followed by Dunnett's T3 test for data with equal variances not assumed. For data with equal variances assumed, ANOVA followed by LSD test was used. A probability of less than 0.05 was considered to be statistically significant.

## 3. Results

### 3.1. Implantation of Embryos on D8

**Figure 1:**
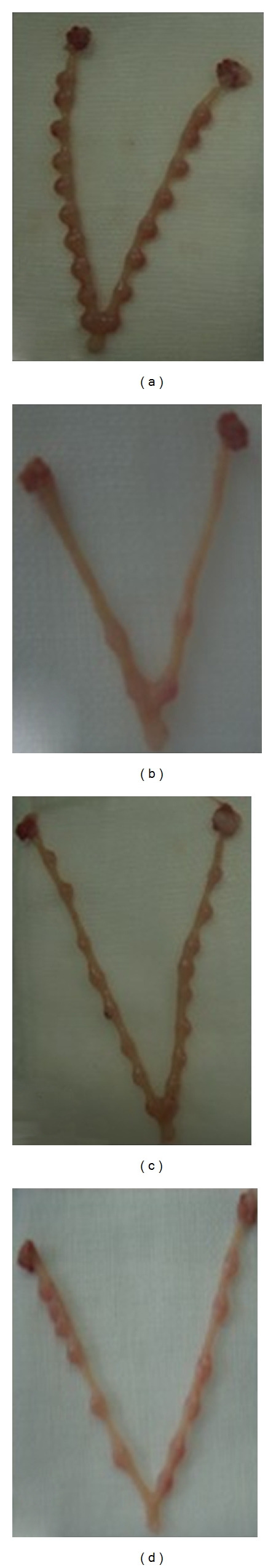
(a) Uterus of group N on D8; (b) uterus of group M on D8; (c) uterus of group A on D8; (d) uterus of group H on D8.

Compared with group N, the number of embryos was significantly lower in group M (*P* < 0.05). In addition, the size of embryos was smaller in group M than in group N. Compared with group M, the number of embryos was significantly higher in groups A and H (*P* < 0.05), and the size of embryos was larger in groups A and H (Figure 1 and Table 1).

### 3.2. Expression of IL-1*β*, IL-2, IL-4, and IL-10 in Serum

Compared with group N, the serum IL-1*β* level was significantly higher in group M on D5 and D6 (*P* < 0.05). And there was a significant reduction in the expression of IL-1*β* in groups A and H when compared with group M on D5 and D6 (*P* < 0.05). Compare with group N, the serum IL-2 level was significantly higher in group M on D6 and D8 (*P* < 0.05), while, compared with group M, there was a significant reduction in the expression of IL-2 in group A on D8 and in group H on D6 and D8 (*P* < 0.05). Compared with group N, the serum IL-4 level was significantly lower in group M on D5 and D6 (*P* < 0.05), while, compared with group M, there was a significant increase in the expression of IL-4 in group A on D5 and in group H on D5 and D8 (*P* < 0.05). Compared with group N, the serum IL-10 level was significantly lower in group M on D6 (*P* < 0.05). And there was a significant increase in the expression of IL-10 in groups A and H when compared with group M on D6 and D8 (*P* < 0.05) (Table 2).

### 3.3. Expression of Endometrial IL-1*β*, IL-2, IL-4, and IL-10 Protein

#### 3.3.1. Outcome of ELISA

The IL-1*β* protein concentration was significantly elevated in group M compared with group N on D5 (*P* < 0.05), while, compared with group M, there was a significant decrease in IL-1*β* protein concentration in group A on D5 and D8 and in group H on D5 (*P* < 0.05). The IL-2 protein concentration was significantly elevated in group M compared with group N on D5 (*P* < 0.05), while, compared with group M, there was a significant decrease in IL-2 protein concentration in groups A and H on D5 (*P* < 0.05). The IL-4 protein concentration was significantly reduced in group M compared with group N on D5, D6, and D8 (*P* < 0.05), while, compared with group M, there was a significant rise in IL-4 protein concentration in groups A and H on D5, D6, and D8 (*P* < 0.05). The IL-10 protein concentration was significantly reduced in group M compared with group N on D5 (*P* < 0.05), while, compared with group M, there was a significant rise in IL-10 protein concentration in groups A and H on D5 (*P* < 0.05) (Table 3).

#### 3.3.2. Outcome of Immunohistochemistry

**Figure 2:**
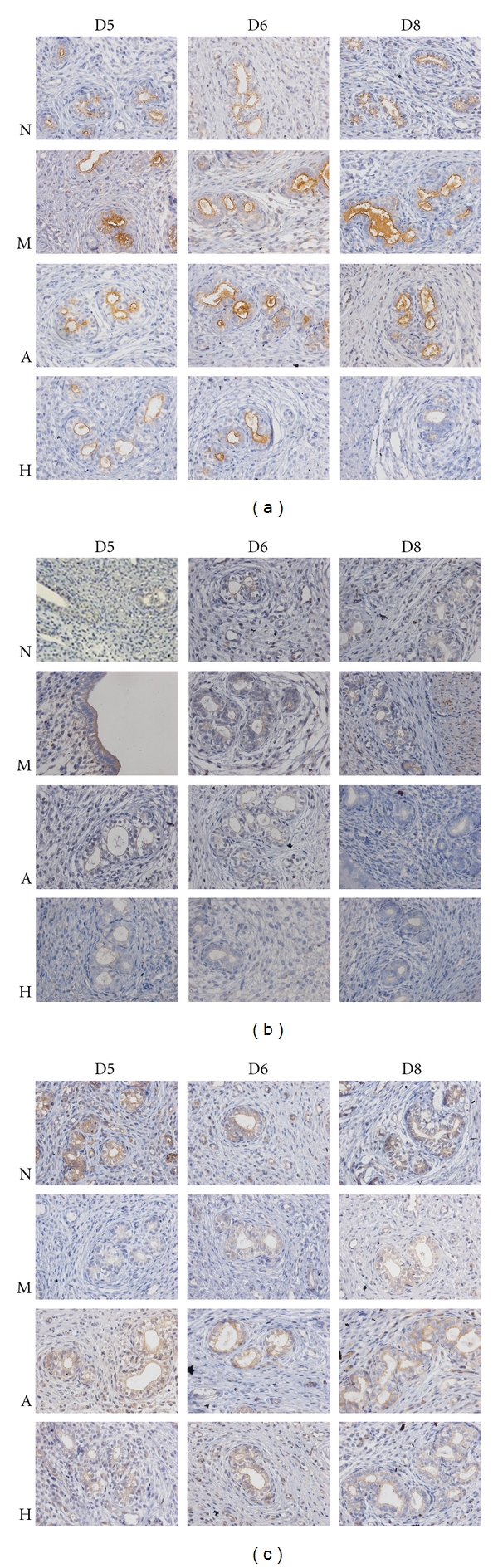
Immunohistochemical staining for the expression of endometrial IL-1*β*, IL-2, and IL-4. Original magnificasion: ×400. Claybank means positive expression. (a) IL-1*β* protein in each group on D5, D6, and D8; (b) IL-2 protein in each group on D5, D6, and D8; (c) IL-4 protein in each group on D5, D6, and D8. IL-1*β*, IL-2, and IL-4 mainly expressed in luminal and glandular epithelium.

**Figure 3:**
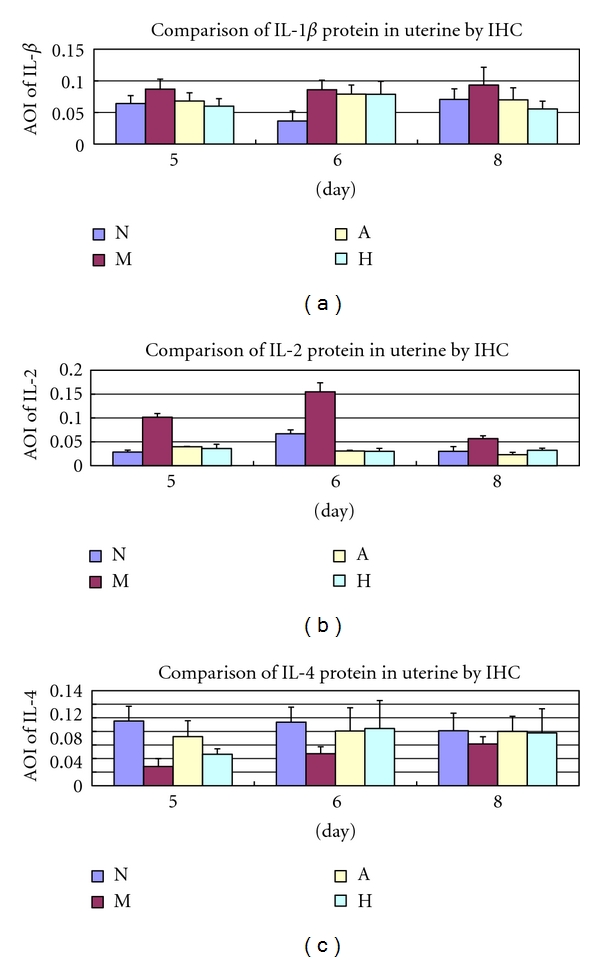
Expression of endometrial protein (a) IL-1*β*; (b) IL-2; (c) IL-4. AOI means average optical intensity. IHC means immunohistochemistry. *represents that there is significant difference when M is compared with N (*P* < 0.05). Δ represents that there is significant difference when A or H is compared with M (*P* < 0.05).

Compared with group N, the endometrial IL-1*β* protein level was significantly increasing in group M on D5, D6, and D8 (*P* < 0.05), while, compared with group M, there was a significant diminution in the expression of IL-1*β* protein in groups A and H on D5 and D8 (*P* < 0.05). Compared with group N, the endometrial IL-2 protein level was significantly increasing in group M on D5, D6, and D8 (*P* < 0.05), while, compared with group M, there was a significant diminution in the expression of IL-2 protein in groups A and H on D5, D6, and D8 (*P* < 0.05). Compared with group N, the endometrial IL-4 protein level was significantly decreasing in group M on D5 and D6 (*P* < 0.05), while, compared with group M, there was a significant elevation in the expression of IL-4 protein in groups A and H on D5 and D6 (*P* < 0.05) (Figures 2 and 3).

#### 3.3.3. Outcome of Western Blot

**Figure 4:**
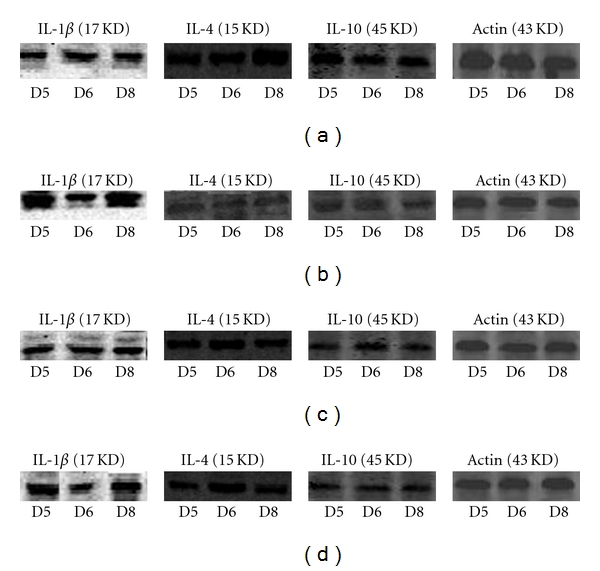
Expression of endometrial IL-1*β*, IL-4, and IL-10 proteins on D5, D6, and D8 in (a) group N; (b) group M; (c) group A; (d) group H.

Compared with group N, the endometrial IL-1*β* protein level was significantly increasing in group M on D5 and D8 (*P* < 0.05), while, compared with group M, there was a significant diminution in the expression of IL-1*β* protein in groups A and H on D5 (*P* < 0.05). Compared with group N, the endometrial IL-4 protein level was significantly decreasing in group M on D5, D6, and D8 (*P* < 0.05), while, compared with group M, there was a significant elevation in the expression of IL-4 protein in groups A and H on D5, D6, and D8 (*P* < 0.05). Compared with group N, the endometrial IL-10 protein level was significantly decreasing in group M on D5, D6, and D8 (*P* < 0.05), while, compared with group M, there was a significant elevation in the expression of IL-10 protein in group A on D5 and D6 and in group H on D5 and D8 (*P* < 0.05) (Figure 4).

### 3.4. Expression of Endometrial IL-1*β*, IL-2, IL-4, and IL-10 mRNA

**Figure 5:**
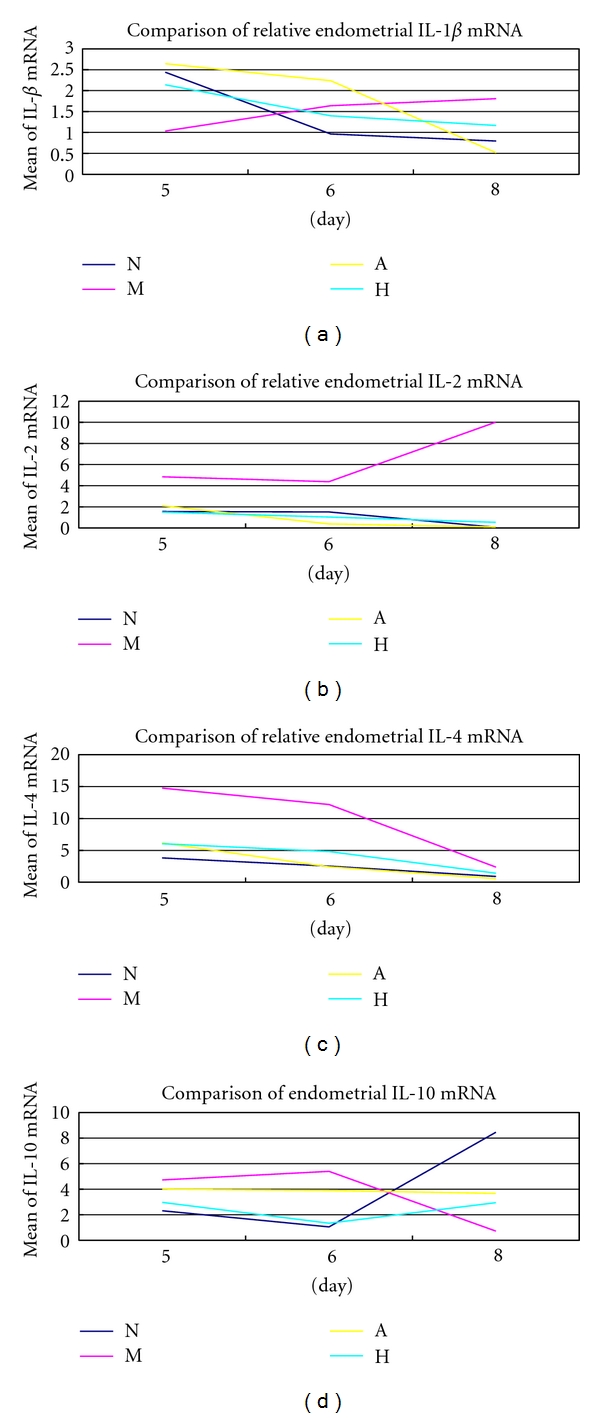
Expression of endometrial mRNA (a) IL-1*β*; (b) IL-2; (c) IL-4; (d) IL-10.

The IL-1*β* mRNA concentration was significantly lower in group M compared with group N on D5 (*P* < 0.05), while, compared with group M, there was a significant increase in IL-1*β* mRNA concentration in groups A and H on D5 (*P* < 0.05). The IL-2 mRNA concentration was significantly elevated in group M compared with group N on D5, D6, and D8 (*P* < 0.05), while, compared with group M, there was a significant decrease in IL-2 mRNA concentration in group A on D5, D6, and D8 and in group H on D5 and D6 (*P* < 0.05). The IL-4 mRNA concentration was significantly increasing in group M compared with group N on D5 and D6 (*P* < 0.05), while, compared with group M, there was a significant drop in IL-4 mRNA concentration in groups A and H on D5, D6, and D8 (*P* < 0.05). The IL-10 mRNA concentration was significantly reduced in group M compared with group N on D8 (*P* < 0.05), while, compared with group M, there was a significant rise in IL-10 mRNA concentration in groups A and H on D8 (*P* < 0.05) (Figure 5).

## 4. Discussion

Mifepristone is the classical antagonist to progestin which has obvious lysis to corpus luteum and resistance to implantation [18]. The development of endometrium was restrained by mifepristone in corpus luteal phase which caused the nonsynchronous growth in endometrium and blastocyst and interfered blastocyst implantation [19]. Mifepristone could blocks progesterone receptors, yet in this study, progesterone counteracted the anti-implantation effect of mifepristone which suggests that excess progesterone could displace the anti-progesterone receptor. Earlier research showed that acupuncture could reverse the implantation resistance of mifepristone, upregulate P level, and significantly increase the average implantation blastocysts number [20]. In the study, the average implantation blastocysts number was notably higher in group A than in group M, which confirmed the effect of acupuncture on antagonizing mifepristone. Acupuncture could improve the poor receptive state of endometrium due to mifepristone, so as to promote the success of blastocyst implantation.

Successful pregnancy has been described as a “Th-2 phenomenon” [14]. Recently, significantly higher serum levels of Th2 cytokines, IL-6 and IL-10 were detected in normal pregnancy compared with unexplained recurrent pregnancy losses and significantly higher serum levels of the Th1 cytokine, IFN-*γ* were present in women with recurrent pregnancy losses, compared with normal pregnancy [21].

IL-2 is a Th1 cytokine that enhances the activity and formation of cytotoxic cells. IL-2 also stimulates TNF*α*, IL-1, and IFN*γ* secretion which are harmful to implantation [22]. The blastocyst implantation of rat occurs on D4 or D5. In this experiment, the expression of endometrial IL-2 mRNA and protein were notably lower in groups N, A, and H than in group M on D5, which verifies that IL-2 is a harmful factor for implantation and demonstrates that acupuncture could effectively reduce the endometrial IL-2 mRNA and protein level and inhibit the cytotoxic effect of IL-2, so as to provide a better environment for blastocysts. However, there is no significant difference among groups in serum IL-2 concentration until D6 or D8. The possible reason might be that IL-2 is mainly produced in uterine during the implantation phase, and there is a delay from secreting in uterine to detecting in serum.

Many events that occur in the fetomaternal interface involve IL-4. IL-4 secreted by endometrium-infiltrating lymphocytes stimulates production of LIF in endometrial tissue [23], which plays a crucial role in implantation and placentation in several species [24]. IL-4 also promotes hCG release in trophoblasts [25], and the released hCG induces production of progesterone from the corpus luteum, which promotes production of Th2 cytokines [26], and displays an antiabortive effect [27]. Our results indicate that endometrial IL-4 protein level was significantly higher in groups N, A, and H than in group M on D5, which is a cue that IL-4 plays a positive role in the process of implantation. At the same time, the results show that acupuncture could promote implantation by elevating the expression of endometrial IL-4 protein. 

IL-10 is one of the cytokines produced by Th2 lymphocytes, whose immunity is necessary for successful pregnancy outcome [8]. IL-10 is an anti-inflammatory cytokine widely present in tissues and fluids during gestation [28], including cyto- and syncytiotrophoblast as well as decidual mononuclear cells/macrophages and NK cells [8, 29, 30]. Decidual monocytes/macrophages and NK cells spontaneously secrete IL-4 and IL-10 in early pregnancy [30]. High levels of IL-10 have also been found in amniotic fluid and maternal serum [8, 27, 31, 32]. IL-10 production was also increased in control women compared with women that were unexplained infertile [33]. A large increase in IL-10 from day 14 to day 2l of pregnancy in women with normal pregnancy outcome compared with those with pregnancy failure was seen in another study [34]. The protection function of IL-10 in implantation is again confirmed in this experiment. Acupuncture could stimulate endometrium to produce IL-10 protein which has resistance to the effect of mifepristone. Serum IL-10 level had a striking increase on D6 in groups N, A, and H compared with group M which is not consistent with endometrial IL-10 level. The delay may be taken into consideration.

In the recurrent implantation failure women, statistically significant elevated levels of IL-1*β* and reduced levels of IL-10 were found [35]. IL-1 stimulates LIF receptor antagonist and decreases implantation rate in the mouse [36]. A more recent study has shown that low plasma concentrations of IL-l*β* and high plasma concentrations of IL-1 Ra are associated with good-quality embryos and increased presence of clinical pregnancies [37]. IL-1*β* negatively affects early stages of pregnancy establishment acting as embryo implantation inhibitors [38, 39]. In addition, Boomsma et al. demonstrated that clinical pregnancy was associated with a significantly lower concentration of IL-1 in endometrial secretions of women [40]. The results of IL-1*β* protein level in this study suggest that IL-1*β* has a deletious effect on implantation as reported in other literatures and also hint that endometrial IL-1*β* protein could be effectively reduced by acupuncture treatment. There is a discrepancy between endometrial mRNA expression and protein level in IL-1*β*, IL-4 which might be coursed by the regulation for protein translation. The high protein level might give a negative feedback to the mRNA expression. Endometrial proteins of IL-4 and IL-10 were highest on D5 in group N, but the peak time for group M was on D6 or even on D8 which prompts that the expression of Th2 cytokines is postponed by mifepristone which retards development of endometrium leading to implantation failure. Though group A reached the height on D6, acupuncture therapy improved the poor receptive state of endometrium by stimulating the expression of Th2 cytokines and reversed the harmful effect of mifepristone to some extent on D5.

In conclusion, we hypothesis that through a series of nerve-physiology-endocrine activities, acupuncture changes the immune microenvironment of endometrium by altering the expression of cytokines in implantation failure uterus including promoting Th2 cytokines such as IL-4 and IL-10 secretion and inhibiting Th1 cytokines including IL-1*β* and IL-2 secretion to improve blastocyst implantation. Next, we have to do the corresponding clinical trials to prove whether acupuncture can produce the same effect in infertile patients. Further rigorous research is needed to confirm the effectiveness of acupuncture.

## Figures and Tables

**Table 1:** Comparison of embryo number on Day 8.

Group	Number of rats	Embryo number
N	6	13.50 ± 2.27
M	6	3.6 ± 1.52*
A	6	9.88 ± 3.14Δ
H	6	11.58 ± 2.64Δ

Value = mean ± SD.

*represents that there is significant difference when M is compared with N (*P* < 0.05).

Δ represents that there is significant difference when A or H is compared with M (*P* < 0.05).

**Table 2:** Comparison of serum cytokines concentration.

Cytokine group	Day	N	M	A	H
IL-1*β*	D5	50.07 ±5.22	76.86 ± 15.35*	50.00 ± 6.81Δ	52.62 ± 7.58Δ
	D6	50.50 ± 3.91	68.26 ± 1.25*	43.65 ± 4.38Δ	45.11 ± 1.84Δ
	D8	27.12 ± 2.77	47.74 ± 9.44	46.57 ± 1.67	42.84 ± 2.83
IL-2	D5	86.13 ± 4.74	90.02 ± 7.03	74.84 ± 15.42	83.05 ± 11.64
	D6	39.46 ± 8.84	61.02 ± 6.66*	57.70 ± 8.15	45.45 ± 7.82Δ
	D8	37.24 ± 0.64	80.14 ± 6.66*	52.69 ± 11.69Δ	54.34 ± 9.05Δ
IL-4	D5	50.67 ± 2.08	16.33 ± 4.72*	43.00 ± 1.00Δ	30.00 ± 5.42Δ
	D6	24.25 ± 2.50	17.00 ± 1.87*	18.40 ± 1.52	20.20 ± 3.63
	D8	25.80 ± 7.26	18.00 ± 2.64	33.67 ± 12.89	44.00 ± 16.00Δ
IL-10	D5	141.87 ± 29.54	84.45 ± 7.35	93.88 ± 14.68	47.19 ± 1.48Δ
	D6	113.07 ± 14.63	34.63 ± 13.02*	207.84 ± 40.27Δ	455.29 ± 37.66Δ
	D8	37.33 ± 7.25	34.37 ± 7.24	69.78 ± 6.94Δ	60.51 ± 3.61Δ

Values are mean ± SD (pg/mL).

*represents that there is significant difference when M is compared with N (*P* < 0.05).

Δ represents that there is significant difference when A or H is compared with M (*P* < 0.05).

**Table 3:** Comparison of endometrium cytokines protein concentration.

Cytokine group	Day	N	M	A	H
IL-1*β*	D5	35.47 ± 2.37	163.73 ± 6.62*	59.03 ± 20.02Δ	95.03 ± 3.36Δ
	D6	52.80 ± 11.90	55.82 ± 14.90	49.13 ± 1.22	46.52 ± 10.4
	D8	74.80 ± 11.75	111.55 ± 9.34	65.57 ± 3.01Δ	71.24 ± 23.91
IL-2	D5	51.78 ± 8.09	79.67 ± 10.65*	60.87 ± 9.83Δ	63.55 ± 12.06Δ
	D6	40.63 ± 0.89	53.44 ± 5.63	44.51 ± 2.15	42.02 ± 3.83
	D8	55.84 ± 3.48	68.81±11.49	66.47 ± 14.63	61.95 ± 12.82
IL-4	D5	189.50 ± 10.61	40.00 ± 10.89*	72.75 ± 23.04Δ	67.00 ± 13.47Δ
	D6	58.33 ± 17.74	34.67 ± 9.50*	74.50 ± 16.05Δ	61.33 ± 7.09Δ
	D8	66.33 ± 3.06	36.00 ± 5.57*	70.00 ± 18.88Δ	76.75 ± 23.04Δ
IL-10	D5	175.79 ± 9.42	127.94 ± 10.25*	185.32 ± 9.39Δ	161.02 ± 15.32Δ
	D6	101.68 ± 21.22	268.95 ± 21.14*	256.29 ± 31.13	264.53 ± 20.55
	D8	119.98 ± 31.77	60.12 ± 1.24	107.89 ± 18.16	126.30 ± 1.84Δ

Values are mean ± SD (pg/mL).

*represents that there is significant difference when M is compared with N (*P* < 0.05).

Δ represents that there is significant difference when A or H is compared with M (*P* < 0.05).
